# ASSESSING THE KNOWLEDGE OF SMALLPOX AND MONKEYPOX VIRUS AMONG THE UNIVERSITY OF TLEMCEN MEMBERS IN THE WAKE OF COVID-19: A 2023 CROSS-SECTIONAL STUDY

**DOI:** 10.21010/Ajidv18i1.2

**Published:** 2023-10-20

**Authors:** ZATLA Ilyes, ABID Wafa, BOUBLENZA Lamia

**Affiliations:** 1 Laboratory of Microbiology Applied to the Food Industry, Biomedical and the Environment, Faculty of Natural and Life Sciences, Earth and Universe Sciences. Department of Biology. University of Tlemcen, Algeria

**Keywords:** Emerging viruses, Monkeypox virus, Monkeypox disease, Public health, Survey, University of Tlemcen

## Abstract

**Background:**

A recently-surfaced virus called Monkeypox virus (MPXV) has gained widespread attention as it dominates the news, creating a sense of panic among people due to the potential threat it poses to their health.

**Materials and Methods:**

To evaluate knowledge about this virus and its disease, and to raise consciousness among the members of the Faculty of Natural and Life Sciences and Earth and Universe Sciences at the University of Tlemcen, we launched an online web-based survey for a twenty days’ period that contained sociodemographic and perceptiveness questions about the emergent virus, its disease, and vaccination.

**Results:**

Our findings showed that the majority of the respondents of our study have a satisfactory level of knowledge about this emerging virus and its disease. Moreover, most participants showed a positive attitude towards the vaccine, considering it the best preventive means to fight against Monkeypox disease.

**Conclusion:**

Although the MPXV may not become a pandemic, but knowing the various ways that contribute to its spread is essential to avoid any possibility of a new one, especially in Algeria.

## Introduction

The emergence and the growing number of Monkeypox virus (MPXV) cases worldwide pose a serious threat to human life, especially in non-endemic countries like Algeria. Additionally, the general poor public understanding of Monkeypox, which makes it a prerequisite for controlling and preventing this zoonotic virus and its disease (Dong *et al.*, 2023). The purpose of our study is to evaluate knowledge of teachers, administrative workers and students of the Faculty of Natural and Life Sciences and Earth and Universe Sciences of University of Tlemcen (NLS/EUS) about this contagious virus in order to promote awareness about the disease and its transmission for anticipating any outbreak in Algeria.

## Materials and Methods

### Type of the study

This study was designed as a cross-sectional web-based survey that took place in the Faculty of NLS/EUS at the University of Tlemcen. Launched for a 20 days period, while having 125 voluntary participants from five different departments of our faculty including Biology, Ecology and Environment, Earth Sciences and Universe, Agronomy, and Forest Resources.

Our survey was created on Google Forms platform in the French language to make it more understandable for the participants of our faculty. It was divided into two different sections, sociodemographic characteristics section and MPXV and disease knowledge’s section, respectively with 25 interrogations, where it was shared through mail and social media platforms such as Facebook.

### Data analysis

The statistical analysis was performed using the statistical software SPSS (Statistical Package for Social Sciences) version 25 and Microsoft Office Excel 2007, as well as the creation of tables and graphs.

## Results and Discussion

In this section, results obtained from the survey were interpreted according to the following criteria.

### Socio-demographic criteria

### Distribution by age and Gender

The results indicate that the majority of respondents were women and belonged the 21-40 years’ age group (46.4%) **(Table 1).** These results may be explained by the fact that younger generations use more of social media than others, and more specifically, females that are interested in learning about the latest developments in the field of health.

**Table 1:** Distribution by Age and Gender

Age	Gender	
	Males	Females
≥20	1.6%	14.4%
21 – 40	5.6%	46.4%
41 – 60	12.8%	18.4%
61– 80	0.8%	0%
Total	20.8%	79.2%

### Criteria on knowledge of Monkeypox virus and its disease

### Distribution according to the knowledge about the causative agent of Smallpox

Results show that most of the participants (90%) previously knew that this disease is related to a virus. These results are consistent with precedents studies carried out in Indonesia (Harapan *et al.*, 2022), Nigeria (Ugwu *et al.*, 2022), China (Ren *et al.*, 2022), and Jordan (Al Mse`adeen *et al.*, 2023).

### Distribution according to the knowledge of the emergence of the new Monkeypox virus

Among the 125 participants, more than two-thirds (61%) have heard about the emergence of the new Monkeypox virus before completing this questionnaire, Youssef *et al.*, (2023) also found the same result in their study from Lebanon. It could be explained by the fact that this virus caused several outbreaks in African and non-African countries so it becomes a topic of news in social media.

### Distribution according to the knowledge of its main sources of transmission

**Figure 1:**
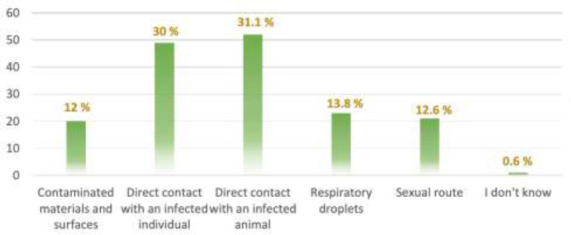
Distribution according to main sources of transmission.

The answers of the respondents about the modes of the Monkeypox virus transmission show that just 47% of acknowledge knowing them. This relatively limited information can be explained by the fact that Algeria has not yet been exposed to an epidemic of this virus. This zoonotic virus can transmit through many routes (Kaler *et al.*, 2022). As a result, all of these transmission routes may be present in combination, for that it is difficult to distinguish between them in any given individual exposure or outbreak scenario (Kwok *et al.*, 2022). In our study we found that among the 66 respondents who declared knowing the sources of transmission of the virus, the majority strongly agreed that Monkeypox could be transmitted through contact with an infected animal or person (31.1%, 30%, respectively) **(Figure 1).**

### Distribution according to knowledge about the rapid transmission of this virus from a person to another

Almost half of participants (43%) did not know if MPXV does spread easily between people or not, and only 39% of them were aware that this virus does spread easily between people, which is in agreement with a previous study conducted in Saudi-Arabia in 2022, where only 41.9% of the participants unanimously agreed on the ease of its transmission between people (Alshahrani *et al.*, 2022). Historically, the virus did not spread efficiently between humans, requiring direct contact with infected body fluids or fomites (bed sheets) or sustained respiratory droplet exposure within 6 meters for 3 hours or more (Ranganath *et al.*, 2022), but recently this emerging virus has proven its great ability to spread among people, and the biggest evidence was in May 2022 where more than over 17,300 confirmed and suspected cases were identified, and over 40.000 infected people in 87 countries that were not MPXV endemic (Gomez-Garberi *et al.*, 2022 ; Hraib *et al.*, 2022).

### Distribution according to the factors that contributed to MPXV emergence

**Figure 2:**
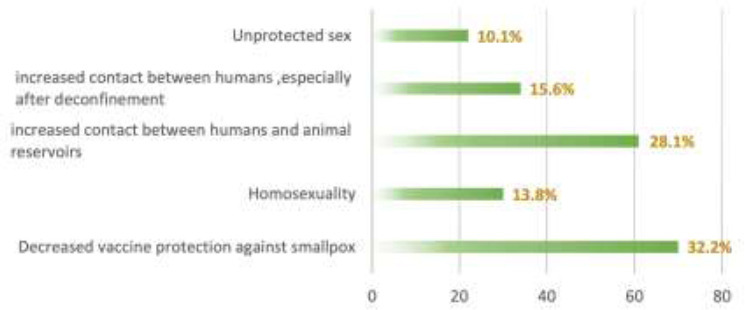
Distribution according to the factors which contributed to its emergence.

According to the participants, there are several factors that promote their emergence and a large part of them (32.2%) consider that the decrease in vaccine protection against smallpox is the first responsible reason for this phenomenon **(Figure 2).** During the 1970s and 1980s, individuals vaccinated against smallpox showed a lower incidence of infection with MPXV, and only 13% of individuals with Monkeypox had a history of smallpox vaccination (Ejaz *et al.*, 2022). Besides, it is very important to note that homosexuality plays a major role in the emergence of this virus. The CDC Data suggest that most patients are amongst the homosexual or bisexual men in the current outbreak (Ajmera *et al.*, 2022).

### Distribution according to the contraction of symptoms associated with this disease

Among the 24 respondents, they indicated that they may be exposed to this disease by carrying associated symptoms, like fever (39.5%), muscle pain and backache (23.2%), skin eruption (23.2%), and swollen lymph nodes (14%). This might be due to the high similarity between the symptoms of Monkeypox, Smallpox, Measles and Chickenpox which may also explain by the fact that Algeria has not yet recorded any official cases of this virus (Altindis *et al.*, 2022 ; Harapan *et al.*, 2022 ; Hraib *et al.*, 2022 ; Titanji *et al.*, 2022).

### Distribution according to the knowledge of preventive measures recommended to avoid transmission of Smallpox disease.

The results show that 48% of the participants knew the preventive measures to avoid transmission of this disease. This result can indicate the consciousness of our participants to follow the news and be updated on the hygienic status concerning emerging viruses in order to avoid repeating the same mistakes made previously in the COVID-19 pandemic era. Whereas according to different studies, there are many preventive measures that can be considered to avoid MPX infection including, avoiding direct contact with animals that are suspected of harboring MPXV, especially in geographical locations where Monkeypox disease is prevalent, to avoid contact with any material that has been in contact with a sick animal or human (Kumar *et al.*, 2022), and to maintain a distance of at least 1 meter from suspected individuals, as well as wearing a well-fitted mask and disposable gloves around them (Ophinni *et al.*, 2022).

Furthermore, cooking all foods containing animal meat or components thoroughly (Upadhayay *et al.*, 2022). On top of that, those with suspected or confirmed MPV infection should isolate themselves at home or at another location covering all lesions, wear a mask around others, wash or disinfect their hands after touching lesions, and not share any personal items (Pastula and Tyler, 2022).

**Figure 3:**
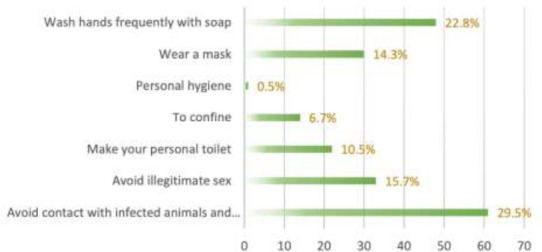
Distribution according to different preventive measures.

In our study, 71 participants have declared that the best ways to prevent the transmission of this virus were to avoid contact with infected animals and sick people, wash hands frequently with soap and avoid illegitimate sexual relations (29.5%, 22.8%, and 15.7%, respectively) **(Figure 3).** Moreover, in a previous study in Jordan, the majority of the respondents agree that good hand hygiene is the best way to prevent this emerging virus (Al Mse`adeen *et al.*, 2023).

### Distribution according to the knowledge of the therapeutic and preventive means against the disease of the Monkeypox

77.3% of the participants knew the therapeutic and preventive means against this disease, and most of them considered that a vaccine is the best way to prevent and treat this disease with 33.3%, followed by antiviral drugs (21.7%). To date, there are no specialized vaccines and antiviral drugs available for MPXV, although other previous vaccines for small Chickenpox viruses, Cidofovir, ST-246, and VIG can be used to control the Monkeypox epidemics (Farasani, 2022). There are categories of people who are more concerned by this treatments including, those whom are suffering from serious diseases such as hemorrhagic diseases, confluent lesions, sepsis, encephalitis, or other diseases requiring hospitalization, also, the immunocompromised population, the pediatric population, especially those under 8 years old, without forgetting pregnant or breastfeeding women, those with a history of allergic dermatitis, and individuals with other active exfoliative skin diseases, in addition to those with one or more complications like, secondary bacterial skin infection ,bronchopneumonia, concurrent diseases, or other co morbidities (Luo and Han, 2022).

### Distribution according to the existence of similarities between infection by the Monkeypox virus and infection by the SARS-CoV-2 virus.

Among all of our participants, 58% indicate that there are no similarities between the two infections, while the rest of them (42%) do distinguish that, there are some common ones (Figure 28). By comparing many studies, we notice that there are some similarities between the two infections in terms of methods of transmission, as both can be transmitted to people through close, direct contact, with infected person’s secretions and droplets, or in terms of the preventive measures that must be taken in order to limit their spread (Zatla *et al.*, 2021a,b ; Kaler *et al.*, 2022). also as both of them share some common symptoms, like, fever, headache and vomiting (Ranganath *et al.*, 2022 ; Zatla *et al.*, 2022)). But we also notice some differences between the two of them like the incubation period which is between 5 and 15 days for SARS-CoV-2 virus and between 5 to 3 weeks for Monkeypox virus, further, the difference in terms of the key symptom, like the classic rash of Monkeypox and ARDS (Acute Respiratory Distress Syndrome) for COVID-19 (Ajmera *et al.*, 2022 ; Zatla *et al.*, 2022).

### Distribution according to the risk of having a new pandemic such as that of SARS-CoV-2

Our data shows that 52% of participants think that there is a risk of having a new pandemic like the one of SARS-CoV-2. However, the WHO director stated that the infectivity of MPXV is relatively low and this virus may not become a pandemic. Despite these allegations, MPX outbreak has become endemic in more than 20 countries since May 2022 (Gong *et al.*, 2022). Adding to that, the difficulty to eradicate this virus since it remerges and exists in multiple animal reservoirs (Weinstein *et al.*, 2005). For that every moment there is a possibility and risk of exposure to this new pandemic.

## Conclusion

In our study which aimed to evaluate knowledge of Teachers, Administrative-Workers and Students of the Faculty of Natural and Life Sciences and Earth and Universe Sciences of University of Tlemcen about Monkeypox virus in order to spread awareness about the disease and its transmission for anticipating any outbreak in Algeria, we found that our participants had a satisfactory level of knowledge for the mode of transmission, and the preventives measures to fight against the spread of this emerging virus. Furthermore, most of the participants showed a positive attitude towards vaccines, considering them as the best therapeutic means to combat the spread of MPX disease. Although there are still many unanswered questions about Monkeypox disease, animal reservoirs, and the virus itself, through this humble work we tried to answer and clarify some important points that all people should know about emerging viruses in general and Monkeypox virus and its disease in particular, in order to prepare in advance for any outbreaks in Algeria.

### Availability of data and materials

The data that support the findings of this study are available from the corresponding author upon request.

### Competing interests

The authors declare no conflict of interest.

**List of Abbreviations:**
MPXV: Monkeypox Virus
MPX: Monkeypox disease
ARDS: Acute Respiratory Distress Syndrome
COVID-19: Coronavirus Disease 2019
SARS-CoV-2: Severe Acute Respiratory Syndrome Coronavirus 2
NLS/EUS: Natural and Life Sciences and Earth and Universe Sciences
